# Patient assessment and feasibility of treatment in older patients with cancer: results from the IN-GHO^®^ Registry

**DOI:** 10.1007/s00432-021-03714-3

**Published:** 2021-07-26

**Authors:** Friedemann Honecker, Susanne Huschens, Ralf Angermund, Gerd Kallischnigg, Werner Freier, Christoph Friedrich, Gerold Hartung, Arnulf Lutz†, Burkhard Otremba, Ludger Pientka, Ernst Späth-Schwalbe, Gerald Kolb, Carsten Bokemeyer, Ulrich Wedding, the Initiative Geriatrische Hämatologie und Onkologie (IN-GHO®).

**Affiliations:** 1 Tumour and Breast Center ZeTuP, St. Gallen, Switzerland; 2 Department of Oncology, Haematology, Bone Marrow Transplantation With Section Pneumology, Hubertus Wald Tumorzentrum, University Medical Center, Hamburg, Germany; 3 Janssen-Cilag GmbH, Neuss, Germany; 4 Argus GmbH, Berlin, Germany; 5 Oncology Practice Hildesheim, Hildesheim, Germany; 6 Department of Geriatrics, St. Maria-Hilf-Krankenhaus, University of Bochum, Bochum, Germany; 7 Oncology Practice Gross-Gerau, Groß-Gerau, Germany; 8 Department of Oncology, University Medical Center Rostock, Rostock, Germany; 9 Oncology Practice Oldenburg, Oldenburg, Germany; 10 Department of Haematology, Oncology, Gastroenterology and Palliative Care, Vivantes Klinikum Spandau, Berlin, Germany; 11 Department of Geriatric and Rehabilitation Medicine, Bonifatius Hospital, Lingen, Germany; 12 Department of Haematology, Oncology, Department of Palliative Care, University Hospital Jena, Am Klinikum 1, 07747 Jena, Germany

**Keywords:** Comprehensive geriatric assessment, Older patients with cancer, Feasibility of treatment, Decision-making, Registry

## Abstract

**Purpose:**

Predicting feasibility of treatment in older patients with cancer is a major clinical task. The Initiative Geriatrische Hämatologie und Onkologie (IN-GHO^®^) registry prospectively collected data on the comprehensive geriatric assessment (CGA), physician’s and patient’s-self assessment of fitness for treatment, and the course of treatment in patients within a treatment decision aged ≥ 70 years.

**Patients and methods:**

The registry included 3169 patients from 93 centres and evaluated clinical course and treatment outcomes 2–3 and 6 months after initial assessment. Fitness for treatment was classified as fit, compromised and frail according to results of a CGA, and in addition by an experienced physician’s and by patient’s itself. Feasibility of treatment (termed IN-GHO^**®**^-FIT) was defined as a composite endpoint, including willingness to undergo the same treatment again in retrospect, no modification or unplanned termination of treatment, and no early mortality (within 90 days).

**Results:**

CGA classified 30.0% as fit, 35.8% as compromised, and 34.2% as frail. Physician’s and patient’s-self assessment classified 61.8%/52.3% as fit, 34.2%/42.4% as compromised, and 3.9%/5.3%, as frail, respectively. Survival status at day 180 was available in 2072 patients, of which 625 (30.2%) had died. After 2–3 months, feasibility of treatment could be assessed in 1984 patients. 62.8% fulfilled IN-GHO**®**-FIT criteria. Multivariable analysis identified physician’s assessment as the single most important item regarding feasibility of treatment.

**Conclusion:**

Geriatricians were involved in 2% of patients only. Classification of fitness for treatment by CGA, and physician’s or patient’s-self assessment showed marked discrepancies. For the prediction of feasibility of treatment no single item was superior to physician’s assessment. However CGA was not performed by trained geriatricians.

**Supplementary Information:**

The online version contains supplementary material available at 10.1007/s00432-021-03714-3.

## Introduction

Cancer is a disease of the older adults. In the United States, more than half of cancer patients are older than 65 years, and about 30% of all cancer deaths occur above the age of 80 (Siegel et al. 2014, 2021). However, due to active exclusion due to trial criteria, or passive non-inclusion by physicians, older patients are underrepresented in clinical trials (Hutchins et al. 1999). Data on feasibility, efficacy, and outcome of treatment in older patients with cancer outside clinical trials are scarce. Decision-making in older patients with cancer can be difficult, as both under- and overtreatment put patients at risk (Pallis et al. 2010a, b). While there are some data on the association of results of a comprehensive geriatric assessment (CGA) with treatment toxicity and mortality, there was limited evidence on how it impacts treatment decision. Recent data show an impact in about 30% of decisions (Hamaker, Te Molder et al. 2018).

Recently, some progress has been made, e.g., by better defining criteria for fitness for treatment, overcoming chronological age as the sole discriminator (Friedrich et al. 2003). A CGA has been advocated as an instrument that can help to identify individual limitations in a multidimensional approach (Maas et al. 2007; Pal et al. 2010; Pallis et al. 2011). Almost 2 decades ago, Hamerman had already gone one step further, and had linked classification according to CGA to treatment decisions (Hamerman 1999). “Fit” patients were deemed fit for standard treatment, whereas “compromised” patients were considered candidates for adapted treatment, and “frail” patients were thought to be largely unfit for cytotoxic treatment. However, until recently, the validity of this or any other classification to guide treatment decisions has, to our knowledge, been examined prospectively only once (Corre et al. 2016).

The “Initiative für Geriatrische Hämatologie und Onkologie” (Initiative for Geriatric Haematology and Oncology, IN-GHO^**®**^) is a working group of German-speaking oncologists and geriatricians exploring clinical aspects of treatment of older patients cancer patients. To this end, the group realized a large prospective registry for older patients with cancer. Besides clinical trials, registries are an important way of collecting data and knowledge on characteristics and outcome of patients with malignancies (Wildiers et al. 2013).

A registry allows collection of data from a clinically relevant subgroup of patients that would otherwise mostly not be taken into consideration. The aims of our registry were to demonstrate feasibility of CGA in the oncological setting, and to identify and analyse clinically important factors for decision-making, including feasibility of treatment. Even though the probability of a good or poor feasibility of treatment is of major importance in clinical decision-making, feasibility of treatment is so far an ill-defined endpoint in oncology (Wildiers et al. 2013, Laurent et al. 2014). To evaluate if treatment decision at baseline was adequate, a combined end-point was defined for feasibility of treatment. To be considered “fit” for the chosen treatment, all of the following criteria had to be fulfilled: (1) during the course of treatment, there was no modification of dose or intensity, and there was no unplanned termination of treatment; (2) the patient did not die within a follow-up period of 90 days (early mortality); and (3) both physician and patient declared at the first assessment at 8–12 weeks, that in retrospect, they would choose the same treatment again (without modifications).

## Patients and methods

Study design and participants: The IN-GHO^**®**^ registry collected data from patients with the following characteristics: age ≥ 70 years, diagnosis of a solid tumour or a haematological malignancy, and a pending treatment decision. This was either start of a new treatment, change of an existing treatment, or even the active decision against cytotoxic therapy. After registration, participating centres, either specialised oncology departments of hospitals (*N* = 22), two of them comprehensive cancer centres (2% = 2/93), or office-based specialised oncologists (*N* = 71), could access the web-based registry. Participating centres were advised to include consecutive patients. An external monitoring was not conducted. All centres were led by board certified oncologists or haematologists. The registry was approved by an institutional review board of the University of Hamburg and informed written consent to collect and analyse pseudonymised data was obtained from each eligible patient before participation. The registry was supported by Janssen-Cilag GmbH.

**Fig. 1:**
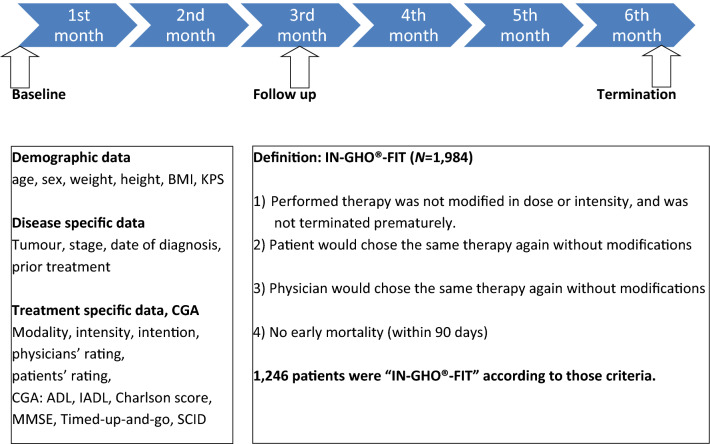
Time schedule of the data collection in the IN-GHO^®^ registry and definition of IN GHO^®^-FIT as a clinical endpoint. Abbreviations: *ADL* activity of daily living, *BMI* body mass index, *CGA* comprehensive geriatric assessment, *IADL* instrumental activity of daily living, *IN-GHO*^*®*^ initiative for geriatric haematology and oncology, *KPS* Karnofsky performance score, *MMSE* mini-mental state examination, *N* number of patients, *SCID* Structured Clinical Interview for DSM-IV screening question

Data were collected prospectively at three different time points, unless observation was terminated prematurely due to patient’s withdrawal of consent, loss to follow-up, or death. Baseline characteristics were documented at inclusion and at two assessment points scheduled during follow-up, first within a window of 8–12 weeks, and again after 6 months (Fig. 1). At baseline, the following data were collected: demographic data (age, sex, weight, body height, body mass index, Karnofsky performance status = KPS), disease-specific information, and recent treatment decision (including modality and intensity of treatment, and palliative or curative intention). Furthermore, physicians, unaware of the results of the geriatric assessment, were asked to subjectively categorize patients’ fitness for treatment into one of three categories (“fit”, “compromised”, or “frail”), and patients’ self-assessment of resilience to stress in categories from 1 (“very resilient”) to 6 (“no resilience”) was documented. Physicians were board certified specialists for internal medicine and haematology/oncology, which included at least a training of 8 years.

### CGA

The CGA included in the first data set at baseline involved the following data and items: assessment of activities of daily living (ADL) (Mahoney and Barthel 1965), instrumental activities of daily living (IADL) (Lawton and Brody 1969), Charlson comorbidity score (Charlson et al. 1987), MMSE (Folstein et al. 1975), timed-up-and-go test (Podsiadlo and Richardson 1991), two screening questions for depression (Structured Clinical Interview for DSM-IV (SCID) depression screening) (Spitzer et al. 1992), co-medication, and history of previous falls. Items of CGA are described in more details elsewhere (Pallis et al. 2010a, b). CGA results were categorized as follows: charlson comorbidity index 0–2 vs. > 2, as most widely practised; comedication 0–3 vs. > 3, as median split; ADL score 100 vs. < 100, as classifying patients without and with limitations, IADL score 8 vs. < 8, as classifying patients without and with limitations; KPS 80–100 vs. < 80, as most widely practised; Timed-up-and-go test, as recommended by the authors; Mini Mental Status Examination 24–30 vs. < 24, as recommended by the authors; SCID as suggested by the manual (Cook et al. 2020, Scheubeck et al. 2021).

### Classification according to CGA

Categorization of older patients with cancer by CGA has been proposed by Balducci and Extermann in 2000 (Balducci and Extermann 2000). Accordingly, we classified our patients as follows: group 1 were independent patients without relevant comorbidity. Group 2 comprised patients with one or two dependencies in IADLs and/or one or two comorbidities, while group 3 comprised frail patients, showing either 1 dependency in ADL, and/or ≥ 3 dependencies in IADL, and/or ≥ 3 comorbidities.

### Feasibility of treatment

To evaluate if treatment decision at baseline was adequate, a combined end-point was defined for feasibility of treatment. To be considered “fit” for the chosen treatment (i.e., to re-assess retrospectively whether an adequate treatment was chosen for the individual patient), all of the following criteria had to be fulfilled: (1) during the course of treatment, there was no modification of dose or intensity, and there was no unplanned termination of treatment; (2) the patient did not die within a follow-up period of 90 days (early mortality); and (3) both physician and patient declared at the first assessment at 8–12 weeks, that in retrospect, they would choose the same treatment again (without modifications). The term “IN-GHO^®^-FIT” was coined for those patients fulfilling all 3 of these criteria.

### Statistical considerations

Descriptive statistics for the overall cohort at baseline were calculated. Patients fulfilling IN-GHO^®^-FIT criteria were compared to patients failing those criteria by Chi squared test for relative data. Stepwise logistic regression models were used to analyse the association of physicians’ and patients’ assessments of fitness or resilience, respectively, and the variables of the CGA with the endpoint IN-GHO^®^-FIT. Inter-rater agreement between assessments was measured by Cohen’s Kappa. The statistical analyses were performed using SPSS 24. To elucidate which pre-therapeutic variables were predictive of IN-GHO^**®**^-FIT, uni- and multivariable analyses were performed including the following variables: sex, age, body mass index, diagnosis, stage of disease, prior tumour surgery, intention of treatment (curative versus palliative), combination versus single-agent therapy, adapted versus standard dose therapy, antibody or hormone treatment, patients’ and physicians’ assessment, and results of the different instruments of the CGA.

## Results

### Patients

**Fig. 2:**
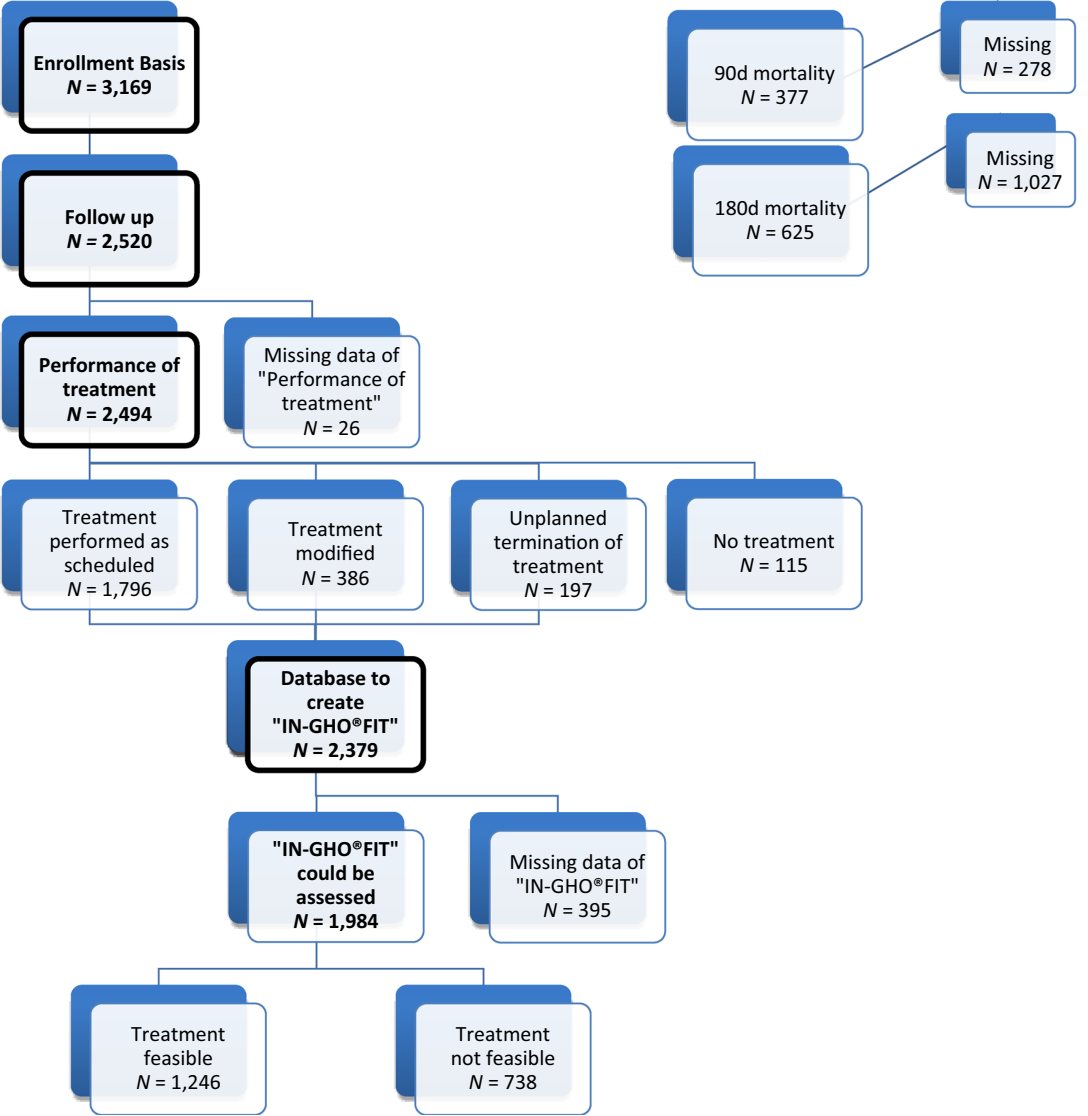
Consort diagram demonstrating availability of patient data in the registry for analyses at different time points of assessment

The consort diagram reports availability of patient data. 3169 patients were included from 2005 to 2011. 47.3% were male, and 23.9% were ≥ 80 years. Mean age was 76.7 years, median age was 75.9 years. 77.9% were treated in an out-patients’ setting, 73.5% had a solid tumour. 63.5% of patients with a solid tumour had metastatic disease. In 54.0% of all patients, first diagnosis of cancer was ≤ 6 months before inclusion into the registry (0–1 month: 24.1%, > 1–6 months: 29.9%). 60.9% of patients had already received prior tumour-specific treatment, either surgery, radiotherapy, chemotherapy, endocrine therapy, or various combinations of those modalities (see supplementary Figure S1). In 49.9% of all patients, an interdisciplinary tumour board was involved in the treatment decision, whereas a geriatrician was involved in only 4.4% of cases. For further details of patients’ characteristics, Table 1 and Fig. 2.

**Table 1:** Baseline characteristics and results of a comprehensive geriatric assessment (CGA)

Baseline characteristics—general		*N*	% or SD
Total cohort		3169	100.0%
Years included	2005–2007	1616	51.0%
	2008–2011	1553	49.0%
Sex	Male	1498	47.3%
	Female	1671	52.7%
Age (years)	70–79	2413	76.1%
	≥ 80	756	23.9%
Age mean/standard deviation (years)		3169	76.7 + / − 4.93
Age median			75.9
BMI (kg/m^2^)	< 19	128	4.1%
	19–< 25	1315	41.9%
	25–< 30	1244	39.6%
	30–< 35	365	11.6%
	35–< 40	69	2.2%
	≥ 40	19	0.6%
Completeness 99%	Missing	29	
BMI mean/standard deviation (kg/m^2^)		3140	25.8 ± 4.3
Main diagnosis	Solid tumour	2329	73.5%
	Haematological Neoplasia	839	26.5%
Completeness 100%	Missing	1	
Diagnosis before baseline (months)	0–1	757	24.1%
	> 1–6	938	29.9%
	> 6–12	249	7.9%
	> 12–60	739	23.5%
	> 60	457	14.6%
Completeness 99%	Missing	29	
Prior tumour-specific treatment	Yes	1930	60.9%
	No	1239	39.1%
Completeness 100%			
Stage (solid tumours *n* = 2329)	Localized	819	36.5%
	Metastastic	1423	63.5%
Completeness 96%	Missing	87	
Diagnosis metastases before baseline			
(Months *n* = 1423)	0–1	368	28.4%
	> 1–6	467	36.0%
	> 6–12	142	10.9%
	> 12–60	283	21.8%
	> 60	37	2.9%
Completeness 91%	Missing	126	
Primary intention of treatment	Curative	861	30.1%
	Palliative	2002	69.9%
Completeness 90%	Missing	306	
Geriatrician was involved	Yes	133	4.4%
	No	2862	95.6%
Completeness 95%	Missing	174	
Interdisciplinary tumour board	Yes	1450	49.9%
	No	1454	50.1%
Completeness 92%	Missing	265	

Baseline characteristics—CGA		*n*	%
Charlson comorbidity score	0–2	2701	89.8%
Range 0–7, median 0	> 2	306	10.2%
Completeness 95%	Missing	162	
Comedication (number of drugs)	0–3	1364	51.3%
Range 0–20, median 3	> 3	1294	48.7%
Completeness 84%	Missing	511	
ADL score	< 100	1170	37.6%
Range 0–100, median 100	100	1944	62.4%
Completeness 98%	Missing	55	
IADL score	< 8	1460	46.6%
Range 0–8, median 100	8	1673	53.4%
Completeness 99%	Missing	36	
Karnofsky performance score (%)	80–100	2119	76.4%
Range 10–100, median 80	< 80	653	23.6%
Completeness 87%	Missing	397	
Timed-up-and-go test	< 10 s	1170	37.8%
Categories reported only	10–20 s	1480	47.8%
	> 20 s/impossible	443	14.3%
Completeness 98%	Missing	76	
Mini mental state examination Score	24–30	2098	81.2%
Range 0–30, median 27	< 24	487	18.8%
Completeness 82%	Missing	584	
SCID screening questions	0 positive	1825	64.8%
Categories reported only	1 positive	654	23.2%
	2 positive	337	12.0%
Completeness 89%	Missing	353	

The numbers of patients in the different categories can be smaller than the number of the total cohort due to missing data. CGA results were categorized as follows: Charlson comorbidity index 0–2 vs. > 2; comedication 0–3 vs. > 3, median split; ADL score 100 vs. < 100 i.e., without and with limitations; IADL score 8 vs. < 8, i.e., without and with limitations; KPS 80–100 vs. < 80; (for details see methods section)

*ADL* activity of daily living; *BMI* body mass index; *CGA* comprehensive geriatric assessment; *IADL* instrumental activity of daily living; *kg* kilogram; *m*^*2*^ square meter, *N* number of patients; *s* seconds; *SCID* Structured Clinical Interview for DSM-IV screening question, thereof the two screening questions for depression; *SD* standard deviation

### Treatment

For 90.3% of patients, primary intention of treatment was captured, which was curative (mostly involving adjuvant therapy) in 30.1%. In 93.9% of patients, the treatment was tumour specific: this was chemotherapy in 86.7%, the rest comprised other treatment modalities (see supplementary Figure S2). 58.8% of patients with chemotherapy received combination and 41.2% single-agent therapy. 81.2% received standard dose and 18.8% dose-adapted treatment (see supplementary Figures S1, S3).

### Assessment

Physicians’ assessment of fitness was as follows: 61.8% of patients were categorized as fit, 34.2% as compromised, and 3.9% as frail. Patients’ self-assessment of resilience to stress was as follows: good and sufficient resilience reported 20.4% and 31.9% of patients, respectively (combined: 52.3%), limited and clearly limited resilience reported 28.4% and 14.0% of patients, respectively (combined: 42.4%), and severely limited resilience or no resilience reported 4.3% and 1.0% of patients, respectively (combined: 5.3%), see supplementary Figure S4. The inter-rater agreement (Cohen’s Kappa) between physicians’ and patients’ assessment was 0.313, which is considered fair (see supplementary Table S1a).

### Follow-up

Follow-up data after 8–12 weeks were available for 2520 patients (79.5% of all 3169 patients). In 72.0% of patients for whom follow-up data were available, treatment was performed as scheduled, in 15.5% it was modified, and in 12.5% it was either not started or there was unplanned termination of treatment. 83.5% of the patients who answered this question stated that they would choose the same treatment again without changes, 9.9% that they would choose it again with modifications, and 6.6% that they would not choose it again; physicians answers were similar, 82.7%, 12.0%, and 5.2%, respectively.

### Rating feasibility of treatment

Furthermore, patients were grouped according to criteria based on CGA (Balducci and Extermann 2000). CGA classified 30.0% as fit, 35.8% as compromised, and 34.2% as frail, see supplementary Figure S4. The inter-rater agreement (Cohen’s Kappa) between CGA and physicians’ and patients’ assessment was 0.100 and 0.151, respectively, which is both considered poor (see supplementary Table S1b + c).

Using IN-GHO^**®**^-FIT criteria, treatment was feasible in 62.8% (1246 patients; data available for 1984 patients). By univariate analysis, the following results showed a significant positive association (*p* < 0.05) with “IN-GHO^**®**^-FIT”: female sex, non-metastatic disease, prior tumour surgery, antibody or hormone treatment, palliative approach, standard dose chemotherapy, both patients’ and physicians’ assessment of fitness or resilience better than compromised/limited, and classification according to Balducci as group 1. From the CGA, the following factors were associated with IN-GHO^**®**^-FIT: Charlson score 0, no dependency in ADL or IADL, KPS ≥ 80%, timed-up-and-go test < 10 s, MMSE score > 24, and negative depression screening by SCID. Tables 2, 3 report the results of the univariate analysis.

**Table 2:** Univariate analysis of association of baseline characteristics with the end-point “IN-GHO^®^-FIT”

Baseline characteristics—general		IN-GHO®-FIT *N* = 1,984: Treatment				*p* value
		Feasible (*N* = 1246)		Not feasible (*N* = 738)		
		*N*	%	*N*	%	
Sex	Male	578	46.4	379	51.4	0.032
	Female	668	53.6	359	48.6	
Age (years)	70–79	972	78.0	553	74.9	0.116
	≥ 80	274	22.0	185	25.1	
BMI kg/m^2^	< 19	41	3.3	31	4.2	0.086
	19< 25	480	38.7	323	43.8	
	25 < 30	526	42.5	290	39.3	
	30 < 35	153	12.3	78	10.6	
	35 < 40	34	2.70	11	1.5	
	≥ 40	5	0.4	4	0.5	
	Missing	7		1		
Main diagnosis	Solid tumour	907	72.8	534	72.4	0.834
	Haematological	339	27.2	204	27.6	
Stage (solid tumours)	Localized	359	41.0	162	31.0	< 0.001
	Metastatic	516	59.0	360	69.0	
	Missing	32		12		
Prior tumour surgery	Yes	505	40.5	256	34.7	0.001
	No	741	59.5	482	65.3	
Intention of treatment	Curative	411	34.8	191	27.4	0.001
	Palliative	771	65.2	507	72.6	
	Missing	64		40		
Chemotherapy modality	Combination	595	59.3	391	60.7	0.558
	Single agent	409	40.7	253	39.3	
	Missing/n.a	242		94		
Dosage (of chemotherapy)	Standard dose	810	85.5	456	74.1	< 0.001
	Dose-adapted	137	14.5	159	25.9	
	Missing	57		29		
Antibody or hormone	Yes	202	16.2	56	7.6	< 0.001
treatment	No	1044	83.8	682	92.4	

Association was considered statistically significant if *p* < 0.05

*N* number of patients; *BMI* body mass index; *n. a.* not applicable

Using a stepwise logistic regression analysis, the following variables were tested for their association with IN-GHO^**®**^-FIT: physicians’ and patients’ assessment, Charlson comorbidity score, KPS, timed-up-and-go test, MMSE, and SCID depression screening. Physicians’ assessment of patients’ fitness was the best parameter in discriminating fit from unfit patients regarding IN-GHO^**®**^-FIT. The resulting logistic regression model (Table 3) was significant (*p* < 0.05). The model made a correct classification in 64.6% of all cases. Interestingly, adding information from the CGA did not help to improve the predictive value regarding feasibility of treatment for the overall cohort (Table 4).

**Table 4:** Univariate analysis: variables of the Comprehensive Geriatric Assessment (CGA) showing significant association (*p* < 0.05) with the end-point “IN-GHO^®^-FIT”

Baseline characteristics—CGA		IN-GHO®-FIT *N* = 1984: treatment				*p* value
		Feasible (*N* = 1246)		Not feasible (*N* = 738)		
		*N*	%	*N*	%	
Physicians assessment of patient fitness	Group 1 (fit)	871	70.4	414	56.6	< 0.001
	Group 2 (compromised)	351	28.4	294	40.2	
	Group 3(frail)	16	1.3	24	3.3	
	Missing	8		6		
Patients’ assessment of resilience to treatment	Good + sufficient	721	59.6	348	49.2	< 0.001
	(Clearly) limited	454	37.6	326	46.1	
	Severely limited + no resilience	34	2.8	33	4.7	
	Missing	37		31		
CGA assessment (Balducci&Extermann)	Group 1 (fit)	415	34.8	190	27.1	0.001
	Group 2 (compromised)	443	37.2	267	38.1	
	Group 3 (frail)	333	28.0	243	34.7	
	Missing	55		38		
Charlson Comorbidity Score	0	656	55.1	355	49.9%	0.043
	1–2	426	35.8	272	38.3	
	> 2	108	9.1	84	11.8	
	Missing	56		27		
ADL Score	100	828	67.3	445	61.5%	0,009
	< 100	402	32.7	279	38.5	
	Missing	16		14		
IADL Score	8	747	60.6	370	50.5	< 0.001
	< 8	485	39.4	362	49.5	
	Missing	14		6		
Karnofsky Performance Score (%)	80–100	926	82.6	509	76.5	0.002
	< 80	195	17.4	156	23.5	
	Missing	125		73		
Timed-up-and-go test	< 10 s	520	42.3	275	38.0	0.010
	10-20 s	580	47.2	342	47.2	
	> 20 s/impossible	128	10.4	107	14.8	
	Missing	18		14		
Mini mental state examination score	24–30	820	84.0	458	80.1	0,048
	< 24	156	16.0	114	19.9	
	Missing	270		166		
SCID screening questions	0 positive	800	70.7	401	60.2	< 0.001
	1 positive	221	19.5	184	27.6	
	2 positive	110	9.7	81	12.2	
	Missing	115		72		

*ADL* activity of daily living; *IADL* instrumental activity of daily living; *IN-GHO®* Initiative for Geriatric Haematology and Oncology; *N* number of patients; *CGA* comprehensive geriatric assessment, *ADL* activities of daily living, *IADL* instrumental acitivies of daily living, *KPS* Karnofsky Performance Status, *s* seconds; *SCID* Structured Clinical Interview for DSM-IV two screening questions

In a second step, we tested different parameters to distinguishing fit from unfit patients in different subgroups of our registry, again using a stepwise regression analysis. Interestingly, in patients with haematological neoplasias (data available for 342 patients), discrimination using the MMSE (cut-off < 24 vs. 24–30) was the only parameter that was significantly (*p* < 0.05) associated with feasibility of treatment besides physicians’ assessment in multivariable analysis, whereas physicians’ assessment remained the only significant parameter in patients with solid tumours (Table 3).

**Table 3:** Stepwise regression analysis for significant variables associated with the end-point “IN-GHO^®^-FIT” in all patients, patients with solid tumours, and patients with haematological malignancies

Cohort		Assessment parameter	Coefficient of regression	Standard error	*p* value	Odds ratio	95% CI	
							Lower	Upper
All patients	*N* = 1236	Physicians’ assessment: fit	0					
		Physicians’ assessment: compromised	− 0.754	0.129	< 0.001	0.470	0.365	0.605
		Physicians’ assessment: frail	− 1.258	0.415	0.002	0.284	0.126	0.641
		Constant	0.852	0.075	< 0.001	2.345		
Patients with solid tumours	*N* = 894	Physicians’ assessment: fit	0					
		Physicians’ assessment: compromised	− 0.637	0.153	< 0.001	0.529	0.392	0.714
		Physicians’ assessment: frail	− 1.527	0.555	0.006	0.217	0.073	0.644
		Constant	0.834	0.087	< 0.001	2.302		
Patients with haematological neoplasia	*N* = 342	Physicians’ assessment: fit	0					
		Physicians’ assessment: compromised / frail*	− 0.903	0.240	< 0.001	0.406	0.253	0.649
		MMSE 24–30	0					
		MMSE < 24	− 0.915	0.312	0.003	0.401	0.217	0.739
		constant	1.016	0.156	< 0.001	2.763		

Of the significant variables (physicians’ and patients’ assessment of fitness, Charlson comorbidity score, ADL, IADL. KPS, timed-up-and-go test, MMSE, and SCID screening questions—compare Table 4), Physicians assessment remained the only significant factor contributing to “IN-GHO^®^-FIT” in all patients and patients with solid tumours. In patients with haematological malignancies MMSE status contributed in addition

*Categories “compromised” and “frail” were combined because of low numbers for category “frail” (*N* = 10)

## Discussion

To our knowledge, we present data from one of the largest prospective registries of older patients with cancer to date. Data were collected from both specialised oncology practices and oncology departments from hospitals. There was a high proportion of patients both > 80 years and with comorbidities or impaired KPS, IADL, or ADL. Even though there is paucity of data regarding the referral practice of older patients with cancer to specialised care (Delva et al. 2012), we believe that this cohort represents a “real world” population of older patients with cancer.

Characteristics of patients enrolled in the registry are similar to a previous report from a German oncology practice (Wedding et al. 2007). Notably, we observed lower rates of geriatric problems compared to a large study from 10 Belgian hospitals, where the rate of patients showing geriatric problems was more than 50% (Kenis et al. 2014). This is most likely due to the higher rate of out-patients in our registry. The 180 days mortality rate in our study was 30.2%, which is in the range of that reported by Arnoldi, with 28.1% (Arnoldi et al. 2007), and Giantin, with 34.4% (Giantin et al. 2013). Soubeyran et al. reported a rate of 16.1%; however, they included patients with first-line treatment only (Soubeyran et al. 2012).

Puts et al. analysed data from studies that examined the impact of a CGA on treatment decisions, the relationship between CGA and toxicity, and correlation of CGA and prediction of mortality (Puts et al. 2012). Several authors identified CGA as a predictor of toxicity (Hurria et al. 2011; Extermann et al. 2012). To our knowledge, factors predicting feasibility of treatment have so far not been reported. Longitudinal reporting of treatment outcome and inclusion of both patients’ and physicians’ evaluation of the chosen treatment allowed us to create a new endpoint termed “IN-GHO^**®**^-FIT”. It consists of willingness to undergo the same treatment again, no need to dose-adapt the chosen treatment, no premature (unplanned) termination, and no early mortality (within 3 months) as a surrogate for treatment futility. We believe that this combined clinical endpoint is helpful in reassessing the initial treatment decision and can help to differentiate adequate treatment from inadequate or futile treatment, which is the most difficult part in caring for older adults with cancer.

Interesting, but somewhat unexpected, we did not find a single tool nor a combination of different tools from the CGA being superior to physicians’ general assessment of fitness for treatment in the prediction of feasibility of treatment (IN-GHO^®^-FIT). We think that this finding might be due to several factors. First, participating oncologists were all experienced clinicians, working in specialised oncology practices or departments, with a certain interest in the management of older patients with cancer. Second, some of the patients were already known to them, as inclusion criteria was not a newly diagnosed cancer but a pending treatment decision. Third, we investigated a very heterogeneous population. Whereas a physician can most likely accommodate to some extent for this heterogeneity by clinical experience, it seems that a single factor or even a combination of several factors is limited in the capacity to deal with this complexity. The finding of the predictive value of the MMSE in the subpopulation of patients with haematological neoplasia, which was not found in the overall population and the subpopulation of patients with solid tumours, could indicate that results might differ in different entities. Our finding is in accordance with two studies that reported cognitive impairment as a strong negative prognostic factor in older patients with haematological neoplasias (Dubruille et al. 2015, Goede et al. 2015). Forth, we did not only include patients at first diagnosis, and therefore many of the patients were already well known to their physicians. As some had already received cancer treatment before by the same physician, one can assume that the physician knew how these patients had fared under the stress of a previous cancer treatment. Fourth, we cannot exclude that the treating physician, who was not blinded to the results of the CGA, might have been influenced by the findings of the tests, thereby “diluting” a possible effect of the CGA.

It will eventually need randomised trials where treatment decision is guided by assessment tools versus physicians’ choice to get a real head-to-head comparison of different discriminators in geriatric oncology (Corre et al. 2016). A retrospective analysis suggests that in diffuse large B-cell lymphoma, CGA might be more accurately identifying patients who benefit from aggressive treatment than clinical assessment (Tucci et al. 2015), and data from a prospective trial in patients with diffuse large B-cell lymphoma indeed show promising results (Spina et al. 2012). Addition data for patients with multiple myeloma (MM) support this (Engelhardt et al. 2020). In a prospective trial in patients with MM Scheubeck et al. identified 4 of the 17 evaluated scores and functional tests as most relevant: the Revised Myeloma Comorbidity Index (R-MCI), Activity of Daily Living (ADL), the Mini-Mental State Examination (MMSE), and the quality-of-life 12-Item Short Form Health Survey Physical Composite Scale (SF-12 PCS) (Scheubeck, Ihorst et al. 2021). On the other hand, none of the studies included in a systematic review by Hamaker et al. used physicians’ assessment of fitness as an assessment tool (Hamaker et al. 2012a, b). In this review, none of the analysed screening method was able to predict impairment in a comprehensive geriatric assessment with sufficient quality.

Against this background, we believe that our data can be interpreted as follows: experienced oncologists are able to correctly choose “adequate treatment” (defined by the IN-GHO^**®**^-FIT criteria) in approximately two-thirds of older patients with cancer. Rather surprisingly, geriatric assessment tools were not able to improve physicians’ assessments in the overall population in this registry. Clearly, more research, possibly also including biological factors, is needed to better discriminate fit from unfit patients in geriatric oncology in the future (Pallis et al. 2013). A systematic review recently analysed the available data regarding the predictive value of a CGA for patients’ outcomes, and concluded that some variables are of predictive value, but the results were still somewhat inconsistent (Hamaker et al. 2012a, b).

Our registry has a number of limitations: (a) data completeness was lacking for this registry, (b) geriatric experts were hardly ever involved, (4.4% of patients), (c) nor did perform CGA, (d) that decision adapted therapy according to physician ratings vs. geriatric tests has not been established so far and/or (e) has not been shown to be necessarily better than wise physician ratings*.* Furthermore, it can be criticized that our definition of “adequate treatment” by the proposed IN-GHO^**®**^-FIT criteria might be adequate in a palliative setting, but less justified in a curative setting or when prolonging overall survival even at the cost of significant toxicity is the ultimate goal.

In conclusion, our study reports several new findings. We propose a novel endpoint, which we term “IN-GHO^**®**^-FIT”, for the assessment of adequate treatment in older patients with cancer. Judgement of patients’ fitness for treatment shows marked discrepancies between rating based on a geriatric assessment, and both physicians’ and patients’ assessment. No single parameter was superior to physician’s assessment in predicting feasibility of treatment. However, even this judgement was correct in only about two-thirds of patients. Interestingly, different subgroups (entities) seem to exist, in which elements of the CGA can contribute relevant information regarding feasibility of treatment. This might indicate the need to develop disease specific assessment tools in oncology/haematology rather than a “one size fits all approach”.

## Supplementary Information

Below is the link to the electronic supplementary material.

Supplementary file1 (DOCX 20 KB)

Supplementary file2 (DOCX 22 KB)

Supplementary file3 (DOCX 35 KB)
