# Supplementary material for: Patient assessment and feasibility of treatment in older patients with cancer: results from the IN-GHO® Registry
Source: J Cancer Res Clin Oncol. 2021 Jul 26;147(11):3183–94. doi: 10.1007/s00432-021-03714-3 (PMC8484105; doi:10.1007/s00432-021-03714-3)
Supplement: Supplementary file 3 — Supplementary file3 (DOCX 35 KB) [file 432_2021_3714_MOESM3_ESM.docx]

Table S1a Inter-rater agreement between physicians’ assessment and patients’ assessment

| **Patient assessment**  **Physician assessment** | **good+sufficient (*N*)** | **(clearly) limited (*N*)** | **severely limited + no resilience (*N*)** | ***N*** | **Kappa** |
| --- | --- | --- | --- | --- | --- |
| **Fit (*N*)** | 1,249 | 581 | 44 | 1,874 | 0.313 |
|  | 41.2% | 19.2% | 1.5% | 61.8% |  |
| **Compromised (*N*)** | 323 | 641 | 79 | 1,043 |  |
|  | 10.7% | 21.1% | 2.6% | 34.4% |  |
| **Frail (*N*)** | 10 | 65 | 39 | 114 |  |
|  | 0.3% | 2.1% | 1.3% | 3.8% |  |
| **Total** | 1,582 | 1,287 | 162 | 3,031 |  |
|  | 52.2% | 42.5% | 5.3% | 100.0% |  |

Table S1b Inter-rater agreement between physicians’ assessment and Balducci rating

| **Physician assessment**  **Rating Balducci** | **Fit (*N*)** | **Compromised (*N*)** | **Frail (*N*)** | ***N*** | **Kappa** |
| --- | --- | --- | --- | --- | --- |
| **Fit (*N*)** | 731 | 158 | 2 | 891 | 0.100 |
|  | 24.6% | 5.3% | 0.1% | 30.0% |  |
| **Compromised** | 713 | 330 | 21 | 1,064 |  |
|  | 24.0% | 11.1% | .7% | 35.8% |  |
| **Frail** | 371 | 550 | 98 | 1,019 |  |
|  | 12.5% | 18.5% | 3.3% | 34.3% |  |
| **Total** | 1,815 | 1,038 | 121 | 2,974 |  |
|  | 61.0% | 34.9% | 4.1% | 100.0% |  |

Table S1c Inter-rater agreement between patients’ assessment and Balducci rating

| **Patient assessment**  **Rating Balducci** | **good+sufficient (*N*)** | **(clearly) limited (*N*)** | **severely limited + no resilience (*N*)** | ***N*** | **Kappa** |
| --- | --- | --- | --- | --- | --- |
| **Fit (*N*)** | 686 | 189 | 6 | 881 | 0.151 |
|  | 23.5% | 6.5% | .2% | 30.2% |  |
| **Compromised (*N*)** | 589 | 433 | 22 | 1,044 |  |
|  | 20.2% | 14.8% | .8% | 35.8% |  |
| **Frail (*N*)** | 228 | 632 | 134 | 994 |  |
|  | 7.8 % | 21.7% | 4.6% | 34.1% |  |
| **Total** | 1,503 | 1,254 | 162 | 2,919 |  |
|  | 51.5% | 43.0% | 5.5% | 100.0% |  |
